# Computed tomography angiography for guiding and follow-up of magnesium-bioresorbable scaffold implantation

**DOI:** 10.1007/s00392-018-1362-8

**Published:** 2018-09-04

**Authors:** Maksymilian P. Opolski, Cezary Kepka, Wojtek Wojakowski, Adam Witkowski

**Affiliations:** 1 Department of Interventional Cardiology and Angiology, Institute of Cardiology, Warsaw, Poland; 2 Department of Coronary and Structural Heart Diseases, Institute of Cardiology, Warsaw, Poland; 3 3rd Department of Cardiology, Upper Silesian Medical Centre, Medical University of Silesia, Katowice, Poland

Sirs:

Numerous studies in the drug-eluting stent era have consistently identified residual stenosis and stent underexpansion as independent predictors of stent restenosis and thrombosis [1, 2]. In this regard, refined techniques for stent implantation with complete coronary lesion coverage and appropriate stent sizing are indispensable to decrease the risk of stent failure and improve outcomes [3, 4]. Although stand-alone coronary angiography often underestimates lesion length and vessel size compared with intravascular ultrasound and coronary computed tomography angiography (CTA), it is still widely used for guidance of percutaneous coronary intervention (PCI) [5]. Recently, the concept of CTA-guided PCI has emerged as a valuable alternative to coronary angiography to provide reliable measurements of the vessel size and lesion length as well as the visualization of the morphological features of coronary plaque [6–8].

A 68-year-old male with atypical angina and a prior history of diabetes mellitus and hypertension was referred for noninvasive coronary imaging. Coronary computed tomography angiography (coronary CTA) using a third-generation dual-source scanner (SOMATOM Definition Force, Siemens Healthcare) showed severe stenosis with low-attenuation plaque, positive remodeling and spotty calcification in the proximal-to-mid portion of the left anterior descending artery (LAD) (Fig. 1a, Online Videos 1 and 2). The interventional treatment strategy (including balloons’ and scaffold’s size) was established according to the measurements of the reference lumen diameters and lesion length on coronary CTA (Fig. 1b). Specifically, the determinant of the nominal scaffold diameter was based on the mean distal reference lumen diameter. Following coronary angiography (Fig. 1c, Online Video 3), predilation with non-compliant high-pressure balloon (Pantera LEO 3.0 × 12 mm at 18 atm) was performed and magnesium-bioresorbable scaffold (Magmaris 3.5 × 20 mm at 14 atm) was implanted in proximal-to-mid LAD (Fig. 1d, e). Finally, the proximal optimization technique using non-compliant balloon (NC Quantum 3.5 × 8 mm at 20 atm) was applied, obtaining optimal angiographic result, along with complete apposition and good expansion of the scaffold in optical coherence tomography (Fig. 1f, g, Online Videos 4 and 5). After 7 months, coronary CTA showed mild atherosclerosis without discernible scaffold struts (Fig. 1h).

**Fig. 1:**
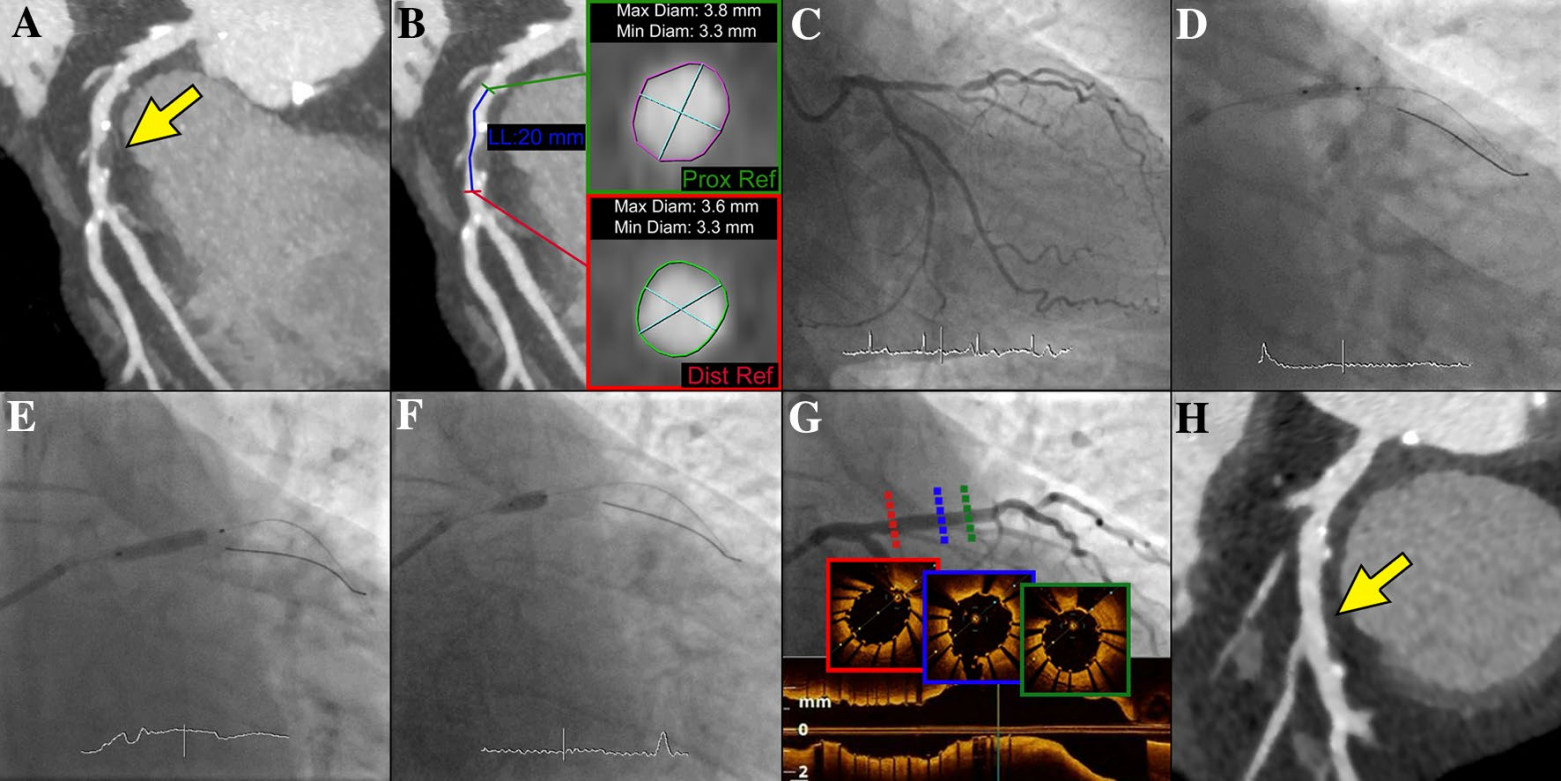
**a** Coronary computed tomography angiography (CTA) disclosing severe stenosis (arrow) in the proximal-to-mid left anterior descending artery (LAD) (Online Videos 1 and 2). **b** CTA-assisted planning of percutaneous coronary intervention (based on computed tomographic data the planned stent length was 20 mm with proximal and distal mean stent diameters of 3.5 mm). **c** Coronary angiography confirming severe stenosis in the proximal-to-mid LAD (Online Video 3). **d** Balloon predilation of the LAD using non-compliant high-pressure balloon (Pantera LEO 3.0 × 12 mm). **e** Magnesium-bioresorbable scaffold (Magmaris 3.5 × 20 mm) implantation in the proximal-to-mid LAD with guidewire protection of the diagonal branch. **f** Proximal optimization technique using non-compliant balloon (NC Quantum 3.5 × 8 mm) in the proximal portion of the scaffold. **g** Final coronary angiography and optical coherence tomography showing optimal angiographic result with complete apposition and good expansion of the scaffold (Online Videos 4 and 5). **h** Coronary CTA demonstrating mild atherosclerosis without discernible scaffold struts (arrow) in the proximal-to-mid LAD at 7-month follow-up

Although intravascular ultrasound is the current gold standard to optimize stent implantation and improve clinical outcomes, there is emerging evidence that measurements of the stent diameter and length performed with coronary CTA and intravascular ultrasound are well correlated [9]. Recently, the concept of CTA-guided PCI has been tested in two randomized trials, wherein CTA-assisted stent sizing resulted in significantly longer stent length and numerically larger stent diameter translating into more complete lesion coverage and less residual disease as compared to angiographic guidance alone [6, 7]. This was corroborated in our case, wherein CTA-assisted PCI resulted in longer and larger scaffold compared to quantitative coronary angiography (Online Fig. 1), and rendered optimal immediate and follow-up results. Furthermore, we believe to show the first-in-man CTA follow-up image of the magnesium scaffold that was not only fully interpretable but also suggested significant bioresorption of the scaffold by 7 months while preserving its luminal integrity. This highlights the potential utility of coronary CTA in guiding and follow-up assessment of bioresorbable scaffolds’ implantation.

## Electronic supplementary material

Below is the link to the electronic supplementary material.

**Fig. 1** Quantitative coronary analysis of the stenosis in the proximal-to-mid left anterior descending coronary artery underestimating the lesion length (11 mm) as well as the proximal and distal lumen diameters (2.68 and 2.88 mm, respectively) as compared to coronary computed tomography angiography (TIF 1475 KB)

**Video 1** Computed tomographic curved multiplanar reconstruction. Curved multiplanar reconstruction showing high-grade lesion with low-attenuation plaque, positive remodeling and spotty calcification in the proximal-to-mid portion of the left anterior descending coronary artery (AVI 3595 KB)

**Video 2** Computed tomographic three-dimensional reconstruction. Three-dimensional reconstruction showing the spatial location of the high-grade stenosis in the left anterior descending coronary artery (AVI 24947 KB)

**Video 3** Baseline coronary angiography. Right anterior oblique caudal view of the coronary angiogram demonstrating severe stenosis in the left anterior descending coronary artery (AVI 12844 KB)

**Video 4** Final coronary angiography. Right anterior oblique caudal view of the coronary angiogram demonstrating good angiographic result after magnesium scaffold implantation (AVI 2228 KB)

**Video 5** Optical coherence tomography. Optical coherence tomography confirming optimal apposition and good expansion of the magnesium scaffold, along with non-flow-limiting proximal edge dissection (AVI 9756 KB)
