# Supplementary figures and images for: Computed tomography angiography for guiding and follow-up of magnesium-bioresorbable scaffold implantation
Source: Clin Res Cardiol. 2018 Sep 4;108(3):344–6. doi: 10.1007/s00392-018-1362-8 (PMC6394473; doi:10.1007/s00392-018-1362-8)

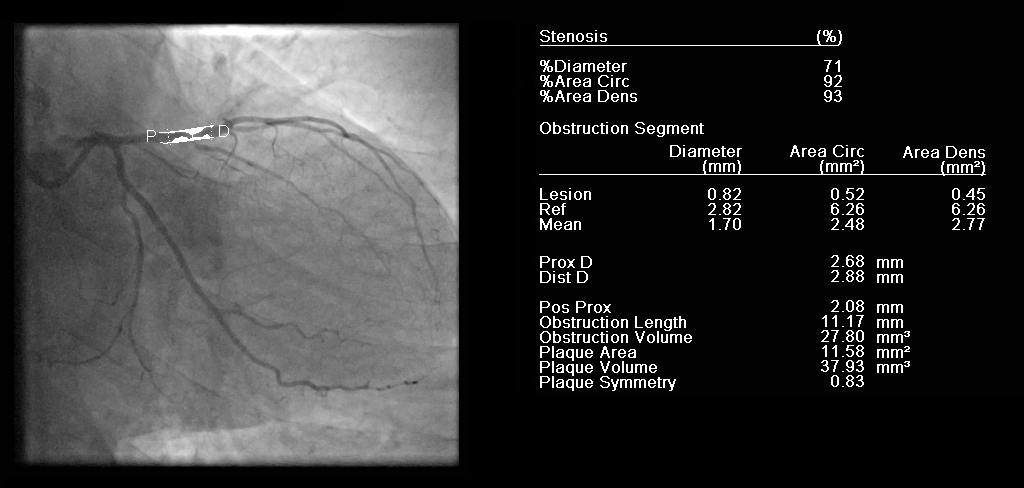

Supplement: Supplementary file 1 — Fig. 1 Quantitative coronary analysis of the stenosis in the proximal-to-mid left anterior descending coronary artery underestimating the lesion length (11 mm) as well as the proximal and distal lumen diameters (2.68 and 2.88 mm, respectively) as compared to coronary computed tomography angiography (TIF 1475 KB) [file 392_2018_1362_MOESM1_ESM.tif]
